# Beyond burnout: A comprehensive investigation of burnout, resilience, and career continuity among Palestinian lawyers in a complex socio-political environment

**DOI:** 10.1371/journal.pone.0310762

**Published:** 2025-01-16

**Authors:** Muayad K. Hattab, Noor Omar Adas, Abdalkarim Ayyoub, Shadi Khalil Abualkibash, Muntaser Nafeth Asmar, Akram Daoud, Ghassan Khaled, Zuheir N. Khlaif

**Affiliations:** 1 Department of Law, Faculty of Law and Political Science, An-Najah National University, Nablus, Palestine; 2 Department of Psychology, Faculty of Humanities and Educational Sciences, An-Najah National University, Nablus, Palestine; 3 Department of Fiqih & Legislation, Faculty of Islamic Law, An-Najah National University, Nablus, Palestine; 4 College of Educational Science, Faculty of Humanities and Educational Sciences, An-Najah National University, Nablus, Palestine; Instituto Federal Goiano, BRAZIL

## Abstract

This article investigates burnout among lawyers and proposes systemic changes to reduce pressure and stress in the legal profession while enhancing resilience among lawyers. The article focuses on factors influencing career continuity among Palestinian lawyers within a socio-politically complex environment. It discusses elements contributing to resilience, including a positive mindset, a strong support system, training, and social support. The study reveals exceptional resilience among Palestinian lawyers. A quantitative cross-sectional study was used; the goals of the study involved 323 participants from different places in Palestine. Data analysis employed Smart PLS4 to build a regression model. Challenging conventional notions, the study provides valuable insights for state strategies, legal firms, educational institutions, and policymakers to address challenges and improve lawyer retention and job satisfaction in an environment marked by unstable economic and political conditions. Additionally, it examines gender-specific career intentions, notably women lawyers displaying a stronger inclination to stay, potentially linked to socio-economic responsibilities. This study contributes significantly to existing literature by providing a unique case study on career continuance determinants within economic and political instability. It sets a precedent for further study and strategic improvements in lawyer retention and job satisfaction within high-stress professions in an economically and politically complex environment.

## 1. Introduction

The legal profession is one of the primary independent professions that, alongside the judiciary, plays a crucial role in achieving justice [1–3] and ensuring the rule of law [4, 5]. The importance of the legal profession and the work of lawyers is not limited to providing their clients with legal advice, but also involves playing an essential, and rather noble role in access to justice, protecting the rights of individuals [6], improving and changing the law and legal rules [7, 8], and even supporting social justice movements [9, 10], and the transformative movement of democracy and equality [8, 11]. This noble mission has been confirmed by Palestinian laws and codes that regulate the legal profession and legal practice [12]. For example, Article (2) of Law No. (3) for the Year 1999 on Regulating Legal Practice, 1999, [13] described the legal profession and advocacy as, “a free profession that assists the judiciary in achieving justice, sustaining the rule of law and ensuring the right to defend the freedoms and rights of citizens”. Furthermore, lawyers and their role has been defined, under Article (2) of the Code of Ethics for the Legal Profession issued by the Palestinian Bar Association (PBA), to include “protectors of rights and defenders of public freedoms, the rule of law, the supremacy of the constitution, the inviolability of the judiciary, the independence of the judiciary, and defenders of national and international justice” [14].

Since the formation of the Palestinian Authority in 1994, the legal profession has received special attention from Palestinian legislator [15]. Lawyers’ practices and conduct are governed by Palestinian Legal Profession Laws issued by the Palestine Legislative Council, Law No. (3) for the Year 1999 on Regulating Legal Practice [13] and Law No. (5) for the Year 1999 on Amendment to the Law on Regulating Legal Practice, [16] or the Palestinian President Decree No. (14) of 2011 [17]. These regulations are complemented by bylaws adopted by the General Assembly of the Palestinian Bar Association P.B.A., of 2000, [18] collectively covering aspects like PBS membership, apprenticeship, qualifications, rights, obligations, ethics, and grievance procedures. To elaborate, in 1997, former Palestinian President Yasser Arafat issued Decree No. 78, establishing for the first time a historic unification of Palestinian lawyers [19]. This decree laid the foundation for the legal profession’s regulation, followed by the issuance of Law No. (3) for the Year 1999 on Regulating Legal Practice by the Palestinian Legislative Council. This law, initially named Regular Lawyers Law No. (3) for the Year 1999, underwent an amendment with Law No. (5) for the Year 1999. Additionally, the Palestinian Bar Association (PBA) adopted the "Lawyers’ Ethics Regulations of 2016" to govern ethical standards in the legal profession [14].

These legal frameworks set forth a structured process for a person to become a practicing lawyer in Palestine. Article (3) of the Law No. (3) on Regulating Legal Practice, 1999 [13] outlines the successive steps, including obtaining a law degree from a recognized university, residency in Palestine, good moral standing, absence of convictions for moral crimes, registration as a trainee lawyer with the Palestinian Bar Association, and completion of legal profession training as specified in the law [20]. Additionally, lawyers in Palestine, just as with most other lawyers in the world [6], are bound by a set of obligations and responsibilities towards the honor of the profession, their clients, and their colleagues. These sets of duties and obligations are primarily regulated in Palestine by the Lawyers’ Ethics Regulations 2016, “the Code of Ethics” [14] and the Law No. (3) for the Year 1999 on Regulating Legal Practice, 1999 [13]. The PBA’s primary focus has been on establishing and upholding a professional code of ethics and addressing occupational concerns [21]. It manages all complaints related to a lawyer’s unprofessional or unethical behavior through its Disciplinary Council. If convicted, the Bar Council may impose penalties such as issuing notifications, reprimands, suspending legal practice for a maximum of five years, or permanently revoking the law license. Any suspension or expulsion from legal practice can be contested in the High Court of Justice.

These regulations are outlined in Articles 33 and 34 of Law No. 3 for the Year 1999, emphasizing the need for lawyers to be aware of and adhere to professional laws and bylaws, prioritizing sincerity, honesty, and dedication to their work [6]. These obligations extend beyond safeguarding clients’ rights to include serving their best interests [22]. Additionally, they require lawyers to maintain a positive image of the profession and competently practice the skills necessary to defend citizens’ rights while upholding ethical standards and preserving professional honor. Article 18 of the Lawyers’ Ethics Regulations of 2016 prohibits lawyers from engaging in any conduct prohibited by Palestinian legislation, either directly or indirectly. Lawyers are also forbidden from accepting employment in other sectors or holding government positions concurrently with their legal practice [14]. Furthermore, Articles 4–20 of the Lawyers’ Ethics Regulations of 2016 forbid lawyers from accepting power of attorney in cases involving conflicts of interest. Lawyers must avoid actions that could harm the public image of their profession, such as soliciting clients, advertising their services, insulting colleagues, breaching client confidentiality, pleading against their clients, or delegating representation without approval [14].

Given the significant role and mission required of lawyers, as well as their efforts to accomplish the challenging tasks imposed on them, their profession can naturally be considered one of the most difficult and stressful. It exposes practitioners to considerable pressure and a heavy burden that must be carried throughout their working lives [23]. Striving to balance professional duties with personal and family commitments becomes a complex challenge, impacting their daily lives [24]. Lawyers also contend with difficult individuals, negative social perceptions, heavy workloads, and challenges to society’s trust in their profession, all of which can influence their performance, decision-making, and relationships [25, 26].

In Palestine, lawyers face additional challenges related to competition within the legal field, exacerbated by a surge in law graduates that has led to widespread unemployment and income disparities [27]. Despite these issues, there are no statistics or substantive research examining the psychological and emotional aspects of the legal profession in Palestine, such as the impact of the profession on lawyers’ mental health, the psychological burnout they experience, or the continuity or interruption of their desire to remain in the profession. The effects on mental health, psychological burnout, and the sustainability of a lawyer’s career remain largely unexplored. Given the immense responsibility, workplace pressure, and limited support, lawyers’ psychological well-being is often negatively impacted [26, 28], underscoring the importance of studying these psychological effects. It has been noted that burnout levels among lawyers are influenced by their workload and the degree of control they have over their work decisions. An increased workload contributes to higher burnout, whereas having more control over decision-making helps to mitigate it [29].

To the best of our knowledge, while much research has been conducted on the topic of burnout, there is limited research on the relationship between burnout and resilience among lawyers [30, 31]. However, the relationship between resilience, burnout, and the continuance intention to remain in the legal profession has not been thoroughly examined and warrants further investigation. To bridge this gap, this study employs a quantitative approach to achieve its objectives. This approach focuses on collecting numerical data using a survey tool, allowing the researcher to investigate the relationships among the factors that influence lawyers to continue working in the legal profession based on their psychological makeup.

Accordingly, this research aims to examine the extent to which the mental health of lawyers is affected by the duties and burdens imposed on them by their profession. It also seeks to explore the relationship between resilience and burnout among lawyers in Palestine, in order to provide accurate conclusions regarding the improvement of their well-being. To achieve these objectives, the following research questions guided the study:

1. How does the legal profession impact the mental health of lawyers, and what are the key factors contributing to burnout and other mental health issues in this field?
2. What measures can be taken to reduce the risk of burnout among lawyers and promote their resilience and well-being?

To address these questions, the article is divided into two main sections. The first section provides a literature review on the relationship between resilience, burnout, and continuance intention among lawyers in Palestine, while the second section introduces the methodology used and presents the results of the study.

## 2. Literature review

As noted earlier, the legal profession imposes significant challenges on lawyers, including time constraints, heavy workloads, intense competition, the need to stay current with diverse legal issues, and the delicate task of balancing personal and professional commitments [32, 33]. Lawyers regularly encounter complex situations characterized by role conflicts, ambiguity, and inconsistency. Notably, courtroom appearances require a delicate balance between the ethical duty to disclose relevant legal authorities, even if detrimental to the client’s position, and the necessity to withhold evidence that may harm the client’s case [34, 35]. These challenges underscore the multifaceted demands and ethical dilemmas inherent in legal practice.

This careful analysis of what should or should not be revealed before the judge can be crucial to the outcome of a case, putting significant pressure on lawyers to carefully consider what they say or present in court [36, 37]. Additionally, lawyers, like any other professionals, face civil liability if their actions or inactions cause harm to a client and are deemed negligent [38]. They may also face disciplinary proceedings if a client files a complaint with the Bar Association for legal malpractice or unethical behavior [39]. If a court or the Bar Association rules against a lawyer, they could be subject to remedies for damages based on negligence and may face suspension from legal practice, revocation of their license, or even permanent removal from the Bar [27, 38].

Conversely, lawyers may find it necessary to hurt the feelings of some litigants or defendants, potentially causing them trauma, during their pleadings or cross-examination in a trial [40]. This becomes particularly sensitive when the case involves children, the elderly, disabled persons, vulnerable women, or any other vulnerable individuals [41]. Lawyers and legal practitioners often represent people during some of the most vulnerable times in their lives, when clients or litigants are likely to experience significant disruption and upheaval during the litigation process and court proceedings [26]. For example, cases involving child custody and divorce, eviction, bankruptcy, or serious criminal allegations such as rape, murder, and homicide are likely to cause trauma and emotional distress to all parties directly involved [42]. Even those indirectly involved, such as close relatives and, in some cases, the wider community, may also experience these emotions [43].

The psychological and emotional impact of these cases is not limited to the incidents themselves but often extends to the legal process. Lawyers, whether representing the plaintiff or the defendant, owe their clients a duty of care and must do all they legally can to help their clients and protect their best interests. This includes questioning statements made by an opponent in a case and challenging the testimony of any supporting witnesses, particularly by highlighting contradictions in evidence and requesting comprehensive details during cross-examination [40]. To reiterate, in cases such as divorce and custody proceedings, emotions can run high, and legal actions involving children often trigger strong feelings of anger, fear, distress, and sadness. While this process serves the interests of a lawyer’s client, it can often cause emotional harm to the opposing party and their family and supporters [44].

On the other hand, the process may also put lawyers under immense emotional stress, potentially causing feelings of guilt and pangs of conscience as they wrestle with the need to do their job effectively while minimizing harm to the feelings of others [45]. Striking the right balance is not an easy task for lawyers and can therefore lead to significant stress [42]. Furthermore, lawyers are particularly at risk of being exposed to emotionally disturbing or distressing material throughout their professional lives, whether during client meetings, litigation, or court proceedings. They are often confronted with statements or evidence containing trauma-exposed content, such as in cases of sexual offenses, child abuse, homicide, human trafficking, slavery, and other criminal, financial, and civil disputes [28, 46]. This repeated and cumulative exposure to trauma-exposed content can cause lawyers emotional distress, potentially leading to trauma-related symptoms [47].

It has been suggested that legal systems should be reformed to include thoughtful education aimed at deepening lawyers’ understanding of the emotional and psychological needs of their clients. This would help them learn effective ways to communicate and work with clients who have personality disorders or who are suffering from trauma or distress [44]. Further, Peña [26], and Ayres [42], have suggested that since lawyers work in a profession that serves vulnerable clients, they should, like medical providers and social workers, receive at least some trauma-informed care training and employ strategies that are mindful of the effects of trauma. While this argument holds value, especially when lawyers are faced with sensitive cases or have vulnerable clients who need compassion, empathy, and emotional support, it should also be extended to address the psychological and emotional well-being of lawyers themselves. This would enable them to work with minimal stress and focus on representing their clients passionately and diligently.

As Colin [28] argued, lawyers must be encouraged to adopt self-care practices, or have trauma-informed policies in place in order to “enhance their well-being and help protect them from work-related trauma but also improve their productivity and efficacy at work” [28, p. 16]. Thus, it is argued that the time has come for the legal profession to incorporate trauma-informed practices into its training, and for lawyers to receive emotion-informed support throughout their professional lives, starting with their education and continuing through their training and legal practice.

### 2.1. Psychological challenges among lawyers

As noted above, the legal profession involves frequent interactions with clients and the meticulous examination of legal matters. Historically, lawyers have been regarded as esteemed defenders of justice, helping clients assert their rights and protecting them from unjust violations. However, they may also be perceived as adversarial opponents by those on the other side of court cases [48]. Such lawyers typically face significant decision-making responsibilities, a high level of self-perceived work stress, and substantial psychological demands in their work [49]. Consequently, the legal profession has been found to have relatively high levels of occupational stress and elevated rates of personal and work-related burnout [48].

The concept of burnout was first introduced in the field of psychosocial literature by Freudenberger [50] and Maslach [51] as a grassroots concept without a theoretical basis. However, it soon evolved into a metaphor for various psychosocial issues faced by individuals who work with people [52]. Today, burnout is widely accepted in psychosocial research and is a well-known term among human service workers in many countries [53, 54]. It is often applied to social workers, nurses, teachers, lawyers, and other white-collar professionals in the human service sector [31].

While individuals in the mental health and social welfare professions are most likely to experience high levels of burnout, research has found that lawyers are also highly susceptible to this problem [55]. This is not solely due to workplace culture and the characteristics of legal practice, but also because of work-related stress, a lack of perceived support from their organizations, and lower levels of empathy within the profession. These findings indicate that stressful and unsupportive working environments can lead lawyers to suffer from stress, emotional distress, and burnout. According to Wallace, lawyers often work long hours and are expected to handle large caseloads with high levels of responsibility and stress. This can contribute to burnout, particularly as the nature of the legal profession requires lawyers to be constantly engaged in intense and demanding work [56].

These factors can significantly impact how lawyers interact with their clients [31]. The demanding expectations and intense competition within the legal field exacerbate the risk of burnout among lawyers. Azeem, Arouj, & Hussain [57], and Tsai, Huang, & Chan [48], highlight that these pressures place lawyers under constant pressure to perform at their best, which, in turn, leads to heightened stress levels and an increased susceptibility to burnout [48, 57]. Moreover, the legal profession is psychologically demanding, and lawyers who experience high levels of psychological demand, effort, and an imbalanced effort-reward ratio are more likely to suffer from both personal and work-related burnout [31].

In a cross-sectional study [48], including 180 lawyers from 26 law firms in Taiwan, it was found that occupational stress was associated with high levels of personal and work-related burnout. Another study revealed that when lawyers’ roles featured high stress, little autonomy, and a cautious mindset that extended to other aspects of their lives, these conditions fostered low spirits and increased burnout, particularly among younger lawyers [58]. Nonetheless, research on burnout [55, 59], has also shown that individuals exhibiting high levels of burnout symptoms are more susceptible to developing cortisol dysregulation, poor job performance, strained relationships, and poorer mental and chronic health. However, other studies have associated burnout with job-related stress and workload [60], suggesting that resilience can help mitigate the negative effects of burnout in various professional settings, including legal and health professions, and prevent poor psychosocial outcomes [61].

### 2.2. Resilience and burnout among lawyers

Resilience is the process of effectively adapting to challenging experiences by demonstrating mental, emotional, and behavioral flexibility, and adjusting to external and internal demands [62, 63]. It involves individuals’ interactions with others, the availability of social resources, and the use of specific coping strategies [64, 65]. Psychological research suggests that positive adaptation, or higher levels of resilience, can be nurtured and developed through cultivation and practice, thereby enhancing the ability to adapt positively to adversity [63, 65]. In the field of positive psychology, resilience is characterized by positive coping and adaptation in the face of significant risk or adversity [66].

When applied in a work setting, resilience, as defined by Luthans [67], refers to the constructive mental strength to recover and adapt in the face of challenges such as adversity, uncertainty, conflict, failure, positive changes, progress, and increased responsibilities. In short, it can be seen as the ability to ’bounce back’ from adversity and continue with life [67]. Studies have shown that individuals can actually become more resilient to adverse situations each time they effectively recover from previous setbacks [68, 69]. Furthermore, research indicates that positive emotions enhance resilience in the face of negative events and can even help individuals surpass their previous baseline levels after experiencing adversity [69]. The level of resilience among employees has been found to be related to their satisfaction, commitment, and happiness [70]. However, resilient employees who have worked in organizations undergoing massive downsizing may struggle to maintain their health, happiness, and performance [71, 72].

Consequently, the more resilience employees can access and utilize to bounce back, the more likely they are to recover from setbacks in the workplace. When combined with hope, resilience provides a clear pathway for bouncing back and moving forward. Although there has been limited research on the relationship between resilience and burnout specifically among lawyers and legal professionals, some studies have demonstrated that resilience is a valuable tool in the legal profession. It can help mitigate the detrimental effects of burnout and promote positive psychosocial outcomes, similar to its impact in other professional domains [73, 74].

### 2.3. The role of resilience in preventing burnout among lawyers

Resilience is a malleable trait that can be enhanced through targeted treatment [75]. Moreover, resilience training offers numerous advantages, such as fostering social competence, problem-solving skills, self-reliance, independence, and a sense of purpose [76]. These resilience-promoting factors have proven beneficial for individuals of all ages, particularly when navigating challenging environmental stressors [77]. Research has revealed that personal strengths, such as hope, optimism, and social support, are significantly associated with reduced burnout, and this relationship is mediated by resilience [78].

To cultivate resilience, it is essential to identify inherent personal strengths that promote an adaptive and positive response to stress. These strengths play a significant role in shaping individual reactions to similar situations, underscoring the importance of recognizing and leveraging them. Research has shown that social support can help prevent burnout [77], and aid lawyers in developing positive coping mechanisms [75, 79], such as positive thinking and problem-solving skills, which can prevent burnout [74, 80].

Another important factor that promotes resilience among lawyers is a positive mindset. Studies have shown that individuals with a positive mindset, optimism, and hope are better able to cope with stress and adapt to change [78, 81]. Furthermore, lawyers who maintain a balance between their work and personal life tend to have lower rates of burnout [82, 83].

### 2.4. Continuance intention among lawyers

Continuance intention has been identified in various studies as a situation in which a person continues to use or pursue an action, they have previously undertaken [84, 85]. In the legal profession, continuance intention among lawyers can be understood as the likelihood or intention of a lawyer, or any individual practicing law, to continue their current behavior or remain engaged in the profession. In this context, continuance intention among lawyers focuses on their inclination to remain in the legal profession and/or continue practicing law.

While several studies have examined the relationship between burnout, resilience, and continuance intention in different professions or contexts—including education, healthcare [84, 86], and marketing—the relationship between these factors among lawyers has not been thoroughly investigated, particularly in Palestine. For example, research in the healthcare field has shown that nurses’ satisfaction and burnout levels were associated with their intention to leave the profession. Factors such as salary, nurse-patient relationships, staffing levels, and the work environment were areas where nurses expressed less satisfaction, whereas group cohesion was associated with greater satisfaction [86]. Similarly, studies on teachers engaged in online instruction found that self-perception of effectiveness and self-efficacy significantly influenced their intention to continue teaching online [84]. Additionally, burnout and technostress were affected by external conditions and personal coping strategies, which in turn influenced teachers’ intentions to utilize digital resources in remote instruction [84, 87].

Harrington et al. [88], found that employees or professionals were more likely to intend to leave their job or profession when experiencing emotional exhaustion, lower levels of intrinsic job satisfaction, and dissatisfaction with salary and promotional prospects. Conversely, factors such as organizational culture, work environment, support from colleagues and superiors, and the availability of resources for professional growth played a significant role in shaping employees’ and professionals’ continuance intention [89, 90]. Moreover, positive workplace conditions—such as a supportive and collaborative atmosphere, opportunities for advancement, healthy work-life integration, and social support—can enhance their commitment and desire to continue in their work or profession [91, 92].

Understanding and addressing the factors that influence continuance intention are crucial for the legal profession to promote the well-being of lawyers, retain personnel in the field, and ensure the overall sustainability of the profession. This is an important issue that warrants further examination through future research using various methods, including interviews and focus groups, to determine the moderating effects of resilience and continuance intention among lawyers.

## 3. Methodology

The researchers in this study employed a quantitative approach to achieve the study’s objectives. This approach focuses on collecting numerical data through the use of a survey tool. Participants were informed about the nature and procedures of the investigation and provided consent before completing the questionnaires. Participation in this study was voluntary. This method enabled the researchers to explore the relationships among the factors that influence lawyers to continue working in the legal profession, particularly in relation to their psychological makeup. Creswell has confirmed that a quantitative design is typically used when researchers aim to conduct statistical analysis [93].

### 3.1. Participants

The participants in this study were 323 practicing lawyers engaged in law and advocacy at various courts across different locations in Palestine. The demographic information of the participants is presented in Table 1. In total, 783 lawyers were contacted, with 412 declining to participate for various reasons, and 48 being excluded due to non-compliance. Consequently, the study includes 323 lawyers from different locations in Palestine who fully participated in the survey. To determine the sample size, it is important to note that, according to data published by the Palestinian Central Bureau of Statistics [94] on August 17, 2023, there are 9,457 practicing lawyers in Palestine. This indicates that our study targeted a sample representing more than 5% of the total lawyer population, with 323 lawyers responding.

**Table 1:** The distribution of the study sample according to independent variables (n = 323).

Independent variables	Levels of variable	Frequency
Gender	Male	195
	Female	128
Experience	Trainer	86
	Less than 5	147
	From 5 to 10	50
	More than 10	37
	Retiree	3
Place	Village	128
	City	195

All procedures performed in this study involving human participants were in accordance with the ethical standards of An-Najah University’s Research Ethics Board (IRB), the American Psychological Association (APA, 2010) and the 2013 Helsinki Declaration and its later amendments or comparable ethical standards. The protocol of our study received ethical approval from An-Najah University’s Research Ethics Board (IRB) under the following number: Ref: Med.D.2023/17. The data collection period spanned nine months, from 25 June 2023 to 29 December 2023. Informed consent was obtained from all participants included in the study. The consent statement explicitly communicated that all information provided is intended for the research study’s nature and will only be used for research purposes.

While we acknowledge that our study may not fully capture the perspectives of lawyers who do not have access to the internet or who chose not to participate in online surveys, we employed multiple distribution methods, including in-person data collection, to enhance the representativeness of our sample. Despite these efforts, we recognize that some degree of selection bias may still exist, which is a common challenge in survey-based research.

### 3.2. Research tool

The Adult Resilience Measure (ARM-R) was used to assess the level of resilience among health care workers (ARM-R) [95]. This scale consisted of 28- items and the responses on it ranged from (1) “strongly disagree” to (5) “strongly agree as rated on a 5-point Likert scale. To interpret the results, means of 5- points Likert scale was utilized as the following: (1–1.80) very low level, (1.81–2.60) low level, (2.61–3.40) moderate level, (3.41–4.20) high level, and (4.21–5) very high level. the use of the ARM-R in the Palestinian context is justified by its cultural adaptability, relevance to the specific challenges faced by Palestinians, and its comprehensive and ethical approach to assessing resilience. It provides valuable data that can inform effective interventions and policies aimed at enhancing resilience and well-being in a conflict-affected population.

Individual burnout will be assessed using the Maslach Burnout Inventory-General Scale 17 items after adapted for Arabic environment [96], which measures three dimensions: emotional exhaustion, depersonalization, and low personal accomplishment. Participants were asked to rate each item on a 7-point Likert scale ranging from 0 (never) to 6 (always) (every day). using the Maslach Burnout Inventory-General Scale in an Arabic context due to its proven validity, reliability, cultural sensitivity, and comprehensive assessment capabilities. This ensures accurate measurement of burnout and facilitates the development of effective interventions tailored to the needs of Arabic-speaking individuals.

In relation to the basis for interpreting mean values under the methodology section, it is important to note that mean values are used to categorize levels of psychological constructs among participants. For instance, emotional exhaustion had a mean of 19.63, indicating a high level, while depersonalization had a mean of 9.38, indicating a moderate level. Interpreting these mean values helps to understand the overall psychological state of the participants. Additionally, for specific scales like the ARM-R (Adult Resilience Measure), a 5-point Likert scale was utilized to interpret the results. The mean scores were categorized into levels such as very low, low, moderate, high, and very high, providing a clearer understanding of participants’ responses based on predefined thresholds. Overall, the interpretation of mean values is grounded in statistical norms for data analysis, with specific benchmarks used to categorize the data into meaningful levels that reflect the psychological constructs being studied.

### 3.3. Translation process

For translation, the researchers used the conceptual equivalence translation method combined with a back-translation approach. Initially, the survey items were translated from Arabic to English by the first translator. The researcher then reviewed these translations for errors and technical inaccuracies, correcting any misunderstandings related to technical and psychological concepts.

Next, the researcher employed a back-translation process by sending the English version to a second translator, who was unaware of the original Arabic content. This translator then translated the text back into Arabic. The researcher compared the back-translated version with the original Arabic content, focusing on conceptual equivalence. This comparison showed a 93% agreement between the translated and original versions. Due to time constraints and the high level of similarity between the translations, a panel of translation experts was not utilized.

### 3.4. Data collection

An online survey (See S1 Appendix) was used to collect data. The researchers designed the survey using Google Form and distributed the link to potential participants. No personal information about the participants was requested. The survey was distributed through various methods: (1) social media, (2) the Palestinian Bar Association, which published the survey on its website, (3) email invitations sent to numerous lawyers, and (4) in-person meetings with lawyers, where the survey was filled out using a tablet.

In total, 783 lawyers were contacted. Of these, 412 declined to participate for various reasons, including busy schedules. Additionally, 48 surveys were excluded because the participants only completed the first part of the survey and did not continue with the rest. The data collection period spanned nine months, from December 28, 2022, to July 25, 2023.

## 3.5. Data analysis

The data was analyzed using the Smart PLS4 statistical package. Before beginning the analysis, the program’s ability to detect missing values and identify the highest and lowest response values for each item ensured that the data was free of outliers and missing values. Consequently, six items containing missing or outlier data were deleted.

The program also handled dummy variables by converting the gender variable values from 1, 2 to 0, 1, and applied similar conversions to the rest of the variables. By selecting the regression mode and inputting the variables, outputs for descriptive statistics, normality, multicollinearity, homoscedasticity, and multiple regression results were automatically generated.

The mean score of 19.63 for emotional exhaustion was derived from the Maslach Burnout Inventory-General Scale (MBI-GS), which consists of multiple items rated by participants on a 7-point Likert scale ranging from 0 (never) to 6 (every day). This scale measures various dimensions of burnout, including emotional exhaustion. The reported mean of 19.63 represents the aggregated scores from multiple items related to emotional exhaustion, rather than a single item score ranging from 0 to 6.

The interpretation of these scores as "high," "moderate," or "low," as shown in Table 2, is based on specific thresholds for each psychological construct being measured. For clarity, when analyzing emotional exhaustion, a total score of 17 or less indicates a low level of burnout, a score between 18–29 represents a moderate level, and a score above 30 is categorized as high burnout. These thresholds are typically established based on prior research or standard scales, such as the Maslach Burnout Inventory, which defines levels of burnout based on aggregate scores from multiple items.

**Table 2:** Means, standard deviations and the level of (Resilience, emotional exhaustion, depersonalization, personal accomplishment, and continuance intention) among lawyers (n = 323).

N	Variables	Mean	SD	Level
1	Emotional Exhaustion	19.63	6.58	High
2	Depersonalization	9.38	7.00	Moderate
3	Personal Accomplishment	9.35	7.51	Moderate
4	Resilience	3.86	.55	Moderate
5	Continuance Intention	3.07	.94	Moderate

*Burnout: Total of 17 or less: low level of burnout

Total between 18–29: moderate level of burnout

Total over 30 high level of burnout

To ensure reliability and validity, we used the Maslach Burnout Inventory-General Scale (MBI-GS) and the Adult Resilience Measure (ARM-R), both of which have established internal consistency (Cronbach’s alpha > 0.70). The instruments were translated into Arabic using the conceptual equivalence method, followed by back-translation. We conducted a pilot study with 30 Palestinian lawyers to test the Arabic version, leading to minor adjustments for cultural and contextual relevance. The Cronbach’s alpha for the Arabic versions was 0.85 for the MBI-GS and 0.82 for the ARM-R, confirming strong internal consistency.

### 3.6. Results

The participants in the study were 323 people from different locations in Palestine who had various experiences. Table 1 provides details of the participants’ distribution based on their demographic information. We noticed that the majority of the participants were living in a city (n = 195; 60%) where around half of the participants had less than five years’ experience in their career.

For data analysis, the normality of the data was checked. Hair et al., [97] suggest the test for normality should be based on skewness and kurtosis to detect for extreme cases of non- normality. Results from the univariate test of normality based on skewness ranged between -1.096 to 0.232 and kurtosis -0.497 to 2.730, indicating some levels of univariate normality but not absolute. This is because the threshold for skewness and kurtosis is between -1 to +1 and -2 to +2 respectively [98, 99]. However, based on Mardia’s coefficient, multivariate normality was not achieved [100]. Results from the multivariate analysis indicated significant p-values for non-normality (Skewness β = 5.065, p<0.01 and kurtosis β = 20.161, p<0.01). However, the results did not indicate extreme non- normality.

Descriptive analysis of the data revealed that emotional exhaustion was high, with a mean of 19.63 and SD 6.58. The levels of the remaining variables were moderate, with variants of mean from 3.07 for continuance intention to continue in the job, to 9.38 for depersonalization. Table 2 summarizes the standard deviation and the means for the variables.

Therefore, we conclude from Table 2 that:

- The level of **Emotional Exhaustion** among lawyers was **High**, as the mean of response for the total score was (**19.63**).
- The level of **Depersonalization** among lawyers was **Moderate**, as the mean of response for the total score was (**9.38**).
- The level of **Personal Accomplishment** among lawyers was **Moderate**, as the mean of response for the total score was (**9.38**).
- The level of resilience among lawyers was **Moderate**, as the mean of response for the total score was (**3.86**).
- The level of **Continuance Intention** among lawyers was **Moderate**, as the mean of response for the total score was (**3.07**).

Multicollinearity, the state in which the independent variables have a high degree of correlation with one another, is crucial. Correlation matrices, whose correlation coefficients should ideally be less than 0.80, or the Variance Inflation Factor (VIF), whose values above 10 indicate serious multicollinearity [101], Table 3 shows that no multicollinearity in our case.

**Table 3:** VIF and correlation.

	VIF	Emotional_Exhaustion	Experience	Personal_Accomplishment	Place	Depersonalization	Gender	Resilience
Emotional Exhaustion	1.85							
Experience	1.05	0.06						
Personal Accomplishment	1.91	0.6	-0.05					
Place	1.06	0.02	0.1	0				
Depersonalization	1.69	0.58	0.04	0.6	0			
Gender	1.1	0.09	-0.12	0.2	0.19	0.07		
Resilience	1.09	-0.15	-0.01	-0.3	0.03	-0.2	-0.02	
Continuance Intention		-0.31	0.05	-0.5	-0.02	-0.2	-0.18	0.35

**Fig 1:**
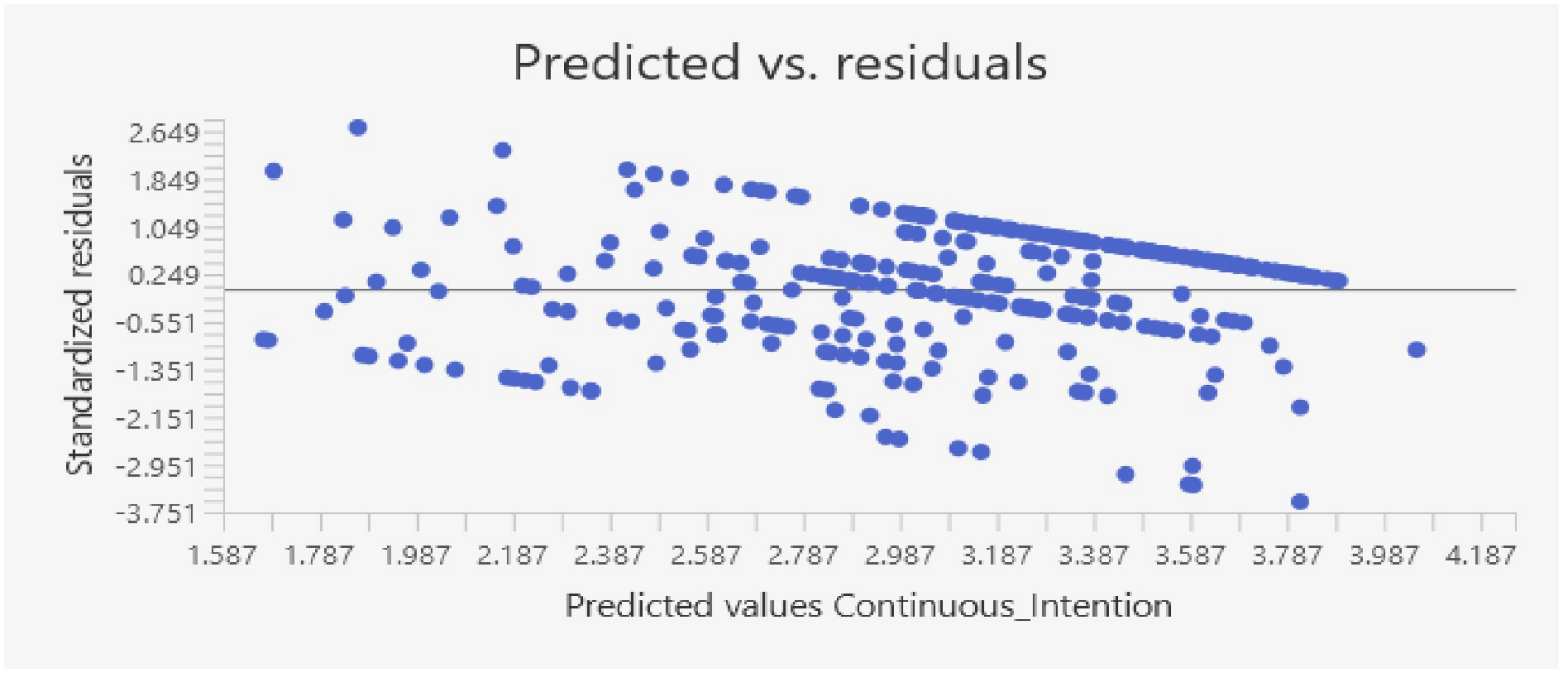
Scatter plot to test homoscedasticity.

Homoscedasticity was accomplished since all levels of the independent variables should have uniform variance for the error terms (residuals). Heteroscedasticity is indicated when there is no clear pattern in a scatterplot of residuals vs anticipated values [102], see Fig 1 below.

**Fig 2:**
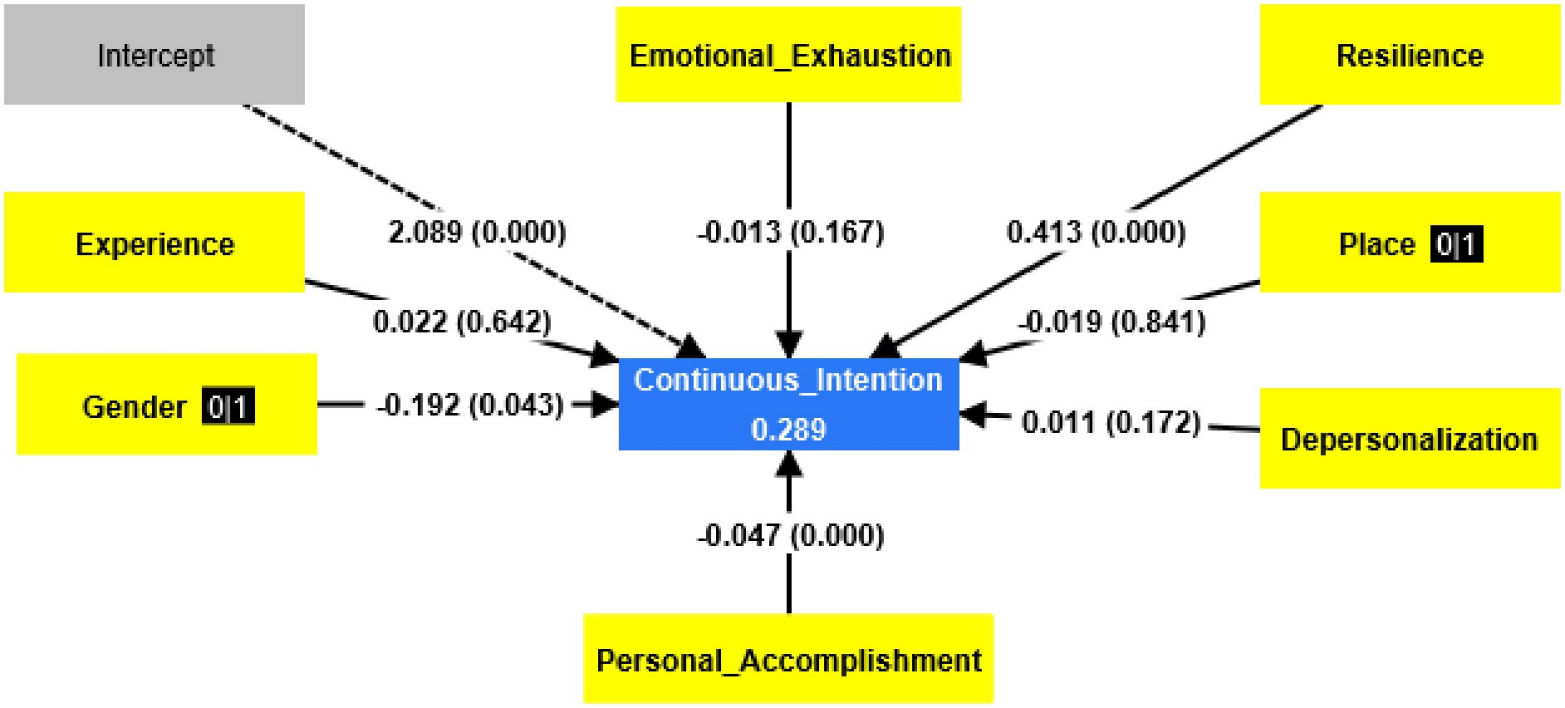
Enter multiple regression.

Through multiple regression analysis, we discovered the relationship among the variables and how these variables influenced the continuance intention of the participants to continue in their jobs. Fig 2 presents the enter multiple regression result among the factors and the continuance intention.

### Unstandardized regression weights (p value)

Based on Fig 2, we conclude that:

1. The model explains nearly.29 of the variances of continuance intention
2. Gender affects (continuance intention) negatively and in favor of ws.
3. Experience does not affect continuance intention.
4. The place of residence does not affect (continuance intention).
5. Emotional exhaustion does not affect continuance intention.
6. Depersonalization does not affect continuance intention.
7. Lack of Personal Accomplishment negatively affects (continuance intention).
8. Resilience positively affects (continuance intention).

## 4. Discussion

The study presents an in-depth analysis of the factors influencing lawyers’ decisions to continue their practice in Palestine, offering a unique perspective on the dynamics of the legal profession in a socio-politically complex region. Notably, it challenges conventional wisdom regarding the role of burnout in career continuance. To achieve the study’s objectives, we employed multiple distribution methods—social media, website postings, email invitations, and in-person data collection—to enhance representativeness.

In total, 783 lawyers were contacted, with 412 declining to participate for various reasons, and 48 being excluded due to non-compliance. Consequently, the study includes 323 lawyers from different locations in Palestine who fully participated in the survey. Despite these efforts, we acknowledge that some degree of selection bias may still exist, which is a common challenge in survey-based research.

Although job burnout is commonly associated with emotional exhaustion and depersonalization, as highlighted in studies by Ersayan et al. [30], and Oberle et al. [103], these factors surprisingly do not deter Palestinian lawyers from continuing their profession. This deviation from expected outcomes suggests a remarkable level of resilience within this group, a perspective supported by Qafisheh [27]. It indicates that despite facing high levels of stress and potential burnout, lawyers in Palestine display a unique determination to persevere in their roles. The study also sheds light on the significant impact of gender on continuance intentions. It finds that gender influences these intentions, particularly among women, aligning with the findings of Shao et al. [104]. Women lawyers in Palestine are more likely to stay in their roles, possibly due to their socio-economic responsibilities, particularly the need to support their families financially, as explored by Bargawi et al. [105]. This gender-specific trend is crucial for understanding the broader socio-economic context that influences career decisions in Palestine, highlighting how socio-cultural norms and economic necessities intertwine to shape professional choices.

Another significant finding of the study is the negligible influence of professional experience and geographical location on career continuity. This contrasts with the results of Panisoara et al. [84], who suggested that these factors are influential in job retention. The unique legal landscape of Palestine, characterized by a surplus of legal graduates leading to intense competition and unemployment, as noted by the Institute of Law [106] and Qafisheh [27], contributes to this divergence. The saturation of the legal market in Palestine creates a context where traditional career influences, such as job experience and location, lose their typical significance in retention decisions. This highlights the distinct challenges faced by lawyers in Palestine, which differ from global trends and require a unique understanding of the region’s legal market dynamics.

Central to the study’s findings is the emphasis on resilience and personal achievement as key factors influencing lawyers’ decisions to continue in their profession. Lawyers who perceive personal success and demonstrate resilience are more inclined to remain, suggesting that these personal attributes are more critical than traditional burnout symptoms in determining career continuity. This aligns with broader discussions on job satisfaction and stress management in career retention, as discussed by Lyng et al., [107] and Brough and Boase [108]. The study underscores the importance of recognizing individual achievements and fostering resilience as strategies for enhancing job satisfaction and retention in high-stress professions.

The study also offers significant practical implications for legal firms, educational institutions, and policymakers in Palestine. The unique challenges and dynamics identified call for targeted strategies to enhance job satisfaction and resilience among lawyers, ensuring a stable and committed legal workforce. For legal firms, this might involve creating more supportive work environments and recognizing individual achievements. Educational institutions could focus on equipping future lawyers with skills to manage stress and build resilience. Policymakers might consider initiatives to address the oversaturation of the legal market and create more equitable job opportunities.

In conclusion, this study provides invaluable contributions to the existing literature, portraying a unique case at the intersection of economic and political instability. The insights gained extend beyond academic discourse, serving as a potential roadmap for policy and organizational changes in the legal sector, especially in regions facing similar challenges. By highlighting the resilience and adaptability of lawyers in Palestine, the study opens avenues for further research and strategic improvements in lawyer retention and job satisfaction, setting a precedent for understanding career intentions in high-stress professions across different socio-political contexts.

### 4.1. Theoretical implications

The current study introduces fresh perspectives on several theoretical implications that can significantly contribute to a broader understanding of career decisions and retention strategies in high-stress professions, particularly in socio-politically complex regions. The study challenges conventional assumptions regarding the influence of burnout on career continuance, suggesting the need to reconsider the significance of burnout symptoms, such as emotional exhaustion and depersonalization, as determinants of career continuation in high-stress professions.

The findings imply the existence of unique stress-coping mechanisms specific to legal practitioners in socio-politically complex environments like Palestine. This suggests that the coping strategies and resilience mechanisms among lawyers in such contexts may differ significantly from those in more stable socio-political settings. The study highlights that, within the context of intensified competition and high unemployment rates among legal practitioners in Palestine, burnout does not significantly diminish the resolve to continue practicing law. This challenges the traditional association between burnout and career decisions, prompting a need for reevaluation within the legal profession.

While gender is a factor in career decisions globally, the study reveals that in the Palestinian context, gender differences in career continuance among lawyers are not as pronounced. The socio-economic circumstances and familial responsibilities in Palestine seem to mitigate this influence on female lawyers’ continuance intentions. This highlights the importance of considering nuanced socio-cultural contexts when studying gender dynamics in career decisions. Moreover, the study identifies the negligible impact of professional experience and geographical location on career continuity in a saturated legal market, redefining established factors influencing job retention. This suggests that in contexts with oversupply and high competition, traditional career influencers might diminish in significance, emphasizing the need for a context-specific understanding of career dynamics.

Furthermore, the study underscores the role of personal achievement and resilience as pivotal determinants of career continuance, highlighting the importance of acknowledging individual successes and fostering resilience as strategies to enhance job satisfaction and retention, particularly in high-stress professions.

### 4.2. Practical suggestions

The study’s findings offer actionable insights for legal firms, educational institutions, and policymakers in addressing the identified challenges. They highlight the need for tailored strategies to enhance job satisfaction and resilience among lawyers, advocating for the creation of more supportive work environments, the integration of stress management skills into legal education, and policy interventions to address the oversaturation in the legal market.

Moreover, the study’s unique findings within a specific socio-political context provide a potential framework for understanding career intentions in similar high-stress professions globally. These insights open avenues for further research to explore the roles of resilience, individual achievement, and context-specific influences on career decisions across diverse socio-political landscapes.

## 5. Conclusion

Resilience and personal achievements emerge as pivotal determinants that significantly influence lawyers’ decisions to persist in their roles, underscoring the imperative for strategies aimed at enhancing job satisfaction and stress management. In exploring the factors influencing Palestinian lawyers’ decisions to remain in their professional roles, this study uncovers unique insights that challenge prevailing notions within the legal profession.

Contrary to established research, it becomes evident that job burnout—typically associated with career discontinuity—does not significantly diminish the resolve of Palestinian lawyers to continue their practice. This unexpected revelation may stem from the intensified competition and soaring unemployment rates prevalent among legal practitioners in Palestine, which create a distinct professional landscape. This finding opens up avenues for deeper investigations into stress-coping mechanisms specific to legal practitioners in socio-politically complex environments.

Conversely, factors such as professional experience and geographical location, often deemed influential in career decisions, do not notably sway continuance intentions among Palestinian lawyers. This peculiarity may be linked to the enduring economic and political instability in Palestine, suggesting the need for further exploration to comprehend the unique dynamics within the legal profession in this context. Additionally, the role of gender in legal career continuance does not substantially affect continuation intentions. While women globally may reconsider their careers due to burnout, Palestinian women lawyers are less likely to be influenced by burnout, likely due to socio-economic circumstances and familial responsibilities.

The study included 323 lawyers practicing in various locations across Palestine, with demographic information provided to address concerns about representativeness. Despite efforts to enhance the sample’s representativeness through multiple distribution methods, we acknowledge that some degree of selection bias may still exist, which is a common challenge in survey-based research. The Adult Resilience Measure (ARM-R) was utilized to assess resilience levels, and the Maslach Burnout Inventory-General Scale was employed to measure burnout dimensions. Results indicated that while emotional exhaustion levels were high, other burnout dimensions and resilience levels were moderate. Notably, emotional exhaustion and depersonalization did not significantly affect the intention to continue practicing law; however, personal accomplishment and resilience did influence continuance intentions.

Multivariate analysis revealed that the model explains nearly 29% of the variance in continuance intention. Gender negatively affected continuance intention, whereas experience and place of residence did not have a significant impact. Emotional exhaustion and depersonalization were also not significant predictors, but personal accomplishment and resilience were.

This study contributes significantly to understanding professional continuance intention by offering unique perspectives from the Palestinian context, marked by enduring economic and political instability. It calls for a paradigm shift in devising job retention strategies within the legal profession, advocating for a nuanced focus on gender roles, recognition of individual achievements, and the fortification of resilience among practitioners. Practical recommendations include creating more flexible and inclusive work environments, especially catering to women, and increasing recognition of lawyers’ accomplishments to bolster their confidence and reinforce their intent to continue in their roles.

## Supporting information

S1 Appendix Survey scales. (DOCX)
