# Supplementary material for: Beyond burnout: A comprehensive investigation of burnout, resilience, and career continuity among Palestinian lawyers in a complex socio-political environment
Source: PLoS One. 2025 Jan 16;20(1):e0310762. doi: 10.1371/journal.pone.0310762 (PMC11737765; doi:10.1371/journal.pone.0310762)
Supplement: S1 Appendix — (DOCX) [file pone.0310762.s001.docx]

# Appendix A: Survey Scales

Questions & Survey Scales

**General Information**

| **Question** | **Options** |
| --- | --- |
| Gender | Male, Female |
| Work Experience | Trainee lawyer, Practicing lawyer < 5 years, Practicing lawyer 5-10 years, Practicing lawyer > 10 years, Retired / Non-practicing lawyer |
| Living Location | City, Village, Camp |

**Occupational Burnout Scale**

| **Question** | **Options** |
| --- | --- |
| I feel like my work is draining me by the end of the day | Never, Once a week, Few times a week, Almost every day |
| I feel emotionally drained because of my profession | Never, Few times a year, Once a month, Once a week, Few times a week, Almost every day |
| I am tired and bored because of my profession | Never, Few times a year, Once a month, Once a week, Few times a week, Almost every day |
| I am frustrated in my profession | Never, Few times a year, Once a month, Once a week, Few times a week, Almost every day |
| I feel like I'm doing my best in my profession | Never, Few times a year, Once a month, Once a week, Few times a week, Almost every day |
| I feel stressed by my direct interactions with people in my profession | Never, Few times a year, Once a month, Once a week, Few times a week, Almost every day |
| I feel that I treat some categories of clients with stagnation and estrangement | Never, Few times a year, Once a month, Once a week, Few times a week, Almost every day |
| I've become a tougher person because of my profession | Never, Few times a year, Once a month, Once a week, Few times a week, Almost every day |
| I worry that my profession will make me harsher and dull my feelings | Never, Few times a year, Once a month, Once a week, Few times a week, Almost every day |
| I don't care what happens to others because of my work | Never, Few times a year, Once a month, Once a week, Few times a week, Almost every day |
| I feel clients blame me for some of the problems they are facing | Never, Few times a year, Once a month, Once a week, Few times a week, Almost every day |
| I feel that I do not have a positive impact on the lives of others through my work | Never, Few times a year, Once a month, Once a week, Few times a week, Almost every day |
| While practicing my profession, I do not feel energetic and lively | Never, Few times a year, Once a month, Once a week, Few times a week, Almost every day |
| I can't create the right atmosphere to do my job to the fullest | Never, Few times a year, Once a month, Once a week, Few times a week, Almost every day |
| While practicing my profession, I do not feel comfortable and happy | Never, Few times a year, Once a month, Once a week, Few times a week, Almost every day |
| I cannot calmly deal with problems and emotional outbursts while doing my profession | Never, Few times a year, Once a month, Once a week, Few times a week, Almost every day |

**Resilience Scale**

| **Question** | **Options** |
| --- | --- |
| I feel that I have not achieved many meritorious things in my profession | Never, Little, Somewhat Little, Frequently, Considerably |
| I have people I can respect in my life and my career | Never, Little, Somewhat Little, Frequently, Considerably |
| I cooperate with lawyers around me | Never, Little, Somewhat Little, Frequently, Considerably |
| Accessing and developing competencies and skills in my profession is important to me | Never, Little, Somewhat Little, Frequently, Considerably |
| I can act in different social situations | Never, Little, Somewhat Little, Frequently, Considerably |
| My family usually helps me in life matters | Never, Little, Somewhat Little, Frequently, Considerably |
| My family knows a lot about me and my profession | Never, Little, Somewhat Little, Frequently, Considerably |
| I try to complete any work related to my profession that I start | Never, Little, Somewhat Little, Frequently, Considerably |
| Spiritual beliefs are a source of strength for me | Never, Little, Somewhat Little, Frequently, Considerably |
| I am proud of my ethnicity | Never, Little, Somewhat Little, Frequently, Considerably |
| People think I'm a fun person for those around me | Never, Little, Somewhat Little, Frequently, Considerably |
| I talk to my family/partner about how I feel | Never, Little, Somewhat Little, Frequently, Considerably |
| I can solve problems without harming myself or others (e.g., without the use of drugs or violence) | Never, Little, Somewhat Little, Frequently, Considerably |
| I feel supported by my friends in my work | Never, Little, Somewhat Little, Frequently, Considerably |
| I know how to get help in my community | Never, Little, Somewhat Little, Frequently, Considerably |
| I feel part of my community through my profession | Never, Little, Somewhat Little, Frequently, Considerably |
| My family stands by me through the difficult times that I am going through in my profession | Never, Little, Somewhat Little, Frequently, Considerably |
| My friends stand by me through difficult times in my profession | Never, Little, Somewhat Little, Frequently, Considerably |
| I am treated normally in my community by virtue of my profession | Never, Little, Somewhat Little, Frequently, Considerably |
| I have opportunities to show others that I can act responsibly | Never, Little, Somewhat Little, Frequently, Considerably |
| I know my strengths | Never, Little, Somewhat Little, Frequently, Considerably |
| I believe it is important to support my community through my profession | Never, Little, Somewhat Little, Frequently, Considerably |
| I feel safe when I am with my family | Never, Little, Somewhat Little, Frequently, Considerably |
| I have opportunities to be useful in life through my profession | Never, Little, Somewhat Little, Frequently, Considerably |
| I enjoy the family and cultural traditions of myself and my family | Never, Little, Somewhat Little, Frequently, Considerably |
| I enjoy the culture of my community | Never, Little, Somewhat Little, Frequently, Considerably |

**Continuing to work in the legal profession**

| **Question** | **Options** |
| --- | --- |
| I intend to continue working in the legal profession in the future | Never, Sometimes, Mostly, Always |
| I plan to continue working in the legal profession | Never, Sometimes, Mostly, Always |
| I expect to continue working in the legal profession in the future | Never, Sometimes, Mostly, Always |
| I will do my best to continue working in the legal profession | Never, Sometimes, Mostly, Always |
